# Xanomeline-Trospium: A Novel Therapeutic for the Treatment of Schizophrenia

**DOI:** 10.1177/10600280251324642

**Published:** 2025-03-27

**Authors:** Olivia L. Ramey, Armando Silva Almodóvar

**Affiliations:** 1 Division of Pharmacotherapy and Translational Sciences, College of Pharmacy, The University of Texas at Austin, Austin, TX, USA; 2 University Health, San Antonio, TX, USA; 3 Long School of Medicine, Pharmacotherapy Education and Research Center, University of Texas Health Science Center, San Antonio, TX, USA; 4 PharmPix Corp., Guaynabo, Puerto Rico

**Keywords:** Schizophrenia, muscarinic agonist, cholinergic agents, serious mental illness, acute psychosis

## Abstract

**Objective:**

This review describes a novel combination muscarinic agonist and antagonist, xanomeline-trospium, which was recently approved by the Food and Drug Administration (FDA) for schizophrenia. Efficacy and safety evidence from phase II and III clinical trials are reviewed.

**Data Sources:**

The MEDLINE and EMBASE databases were searched in 2024; terms included “xanomeline trospium” OR “xanomelinetrospium” OR “KarXT” OR “Cobenfy” AND “schizophrenia.” The search was repeated in January 2025. Clinicaltrials.gov was used to review ongoing or unpublished studies.

**Study Selection and Data Extraction:**

Human subject studies of xanomeline-trospium were included.

**Data Synthesis:**

In phase III trials, xanomeline-trospium was superior to placebo for acute exacerbation of schizophrenia. EMERGENT-2 and EMERGENT-3 found patients improved by 9.6 and 8.4 points on the Positive and Negative Symptom Scale (PANSS), respectively, compared with placebo (95% confidence interval –13.9 to –5.2, *P* < 0.001 and –12.4 to –4.3, *P* < 0.001). Participants in EMERGENT-4, a long-term open-label extension study, who received the intervention in the randomized phases experienced a 9-point decrease in PANSS from the last result.

**Relevance to Patient Care and Clinical Practice in Comparison to Existing Drugs:**

Xanomeline-trospium is a novel antipsychotic that is effective for treatment of schizophrenia and may have a favorable adverse effect profile in comparison with other antipsychotics due to its lack of dopamine receptor antagonism. Efficacy for improvement in negative symptoms and cognitive function in schizophrenia is promising.

**Conclusions:**

Xanomeline-trospium shows promising results for treatment of schizophrenia. Further studies with active comparators, larger sample sizes, and more patients with prominent negative symptoms are needed to corroborate efficacy and determine place in therapy.

## Introduction

Schizophrenia is a serious mental health condition affecting 24 million people worldwide.^1^ Schizophrenia is characterized by disruptions in thought processes or beliefs, emotional responsiveness, and social function.^1^ Worldwide, schizophrenia is among the leading causes of disability and is associated with premature mortality, as well as decreased engagement and retention in the health care system.^1^ Among people with schizophrenia, the rate of death by suicide (4.9%) is much higher than the general population rate of death by suicide, which was 0.014% in 2021.^1,2^ The clinical presentation of schizophrenia is complex, and includes positive, negative, and cognitive symptoms.^3^

The American Psychiatric Association (APA) published an updated guideline on the treatment of schizophrenia in 2020.^4^ The APA guideline recommends treatment with an antipsychotic medication and monitoring for efficacy and adverse effects based on a high level of evidence.^4^ Unfortunately, antipsychotic medications have potentially serious adverse effects: metabolic syndrome, hyperprolactinemia, QT prolongation, extrapyramidal symptoms (EPS), and neuroleptic malignant syndrome (NMS).^4^ Adverse effects are highly associated with nonadherence to treatment, which is known to diminish treatment response.^4^ In addition, antipsychotic pharmacotherapy is most efficacious in treatment and prevention of positive symptoms, with a lesser effect on potentially debilitating negative and cognitive symptoms.^4^ These symptom domains can cause people with schizophrenia to suffer from continued problems with social functioning, maintaining employment, and other executive function tasks despite antipsychotic treatment.^5^

Receiving Food and Drug Administration (FDA) approval on September 26, 2024, xanomeline-trospium (Cobenfy)^6^ is a first-in-class medication for treatment of schizophrenia, based on placebo-controlled trials in the acute care setting that examined the treatment as monotherapy. The purpose of this new drug review is to examine safety and efficacy data from clinical trials of xanomeline-trospium.

## Data Sources

The MEDLINE and EMBASE databases were searched using the following terms: “xanomeline trospium” OR “xanomelinetrospium” OR “KarXT” OR “Cobenfy” AND “schizophrenia.” ClinicalTrials.gov was used to identify ongoing clinical trials.

## Study Selection and Data Extraction

The phase I pharmacokinetic and pharmacodynamic study was reviewed.^7^ Phase II and III studies were included in description of clinical safety and efficacy data in the treatment of schizophrenia.^8-10^ Open-label extension studies were identified, and non-peer reviewed results are included as available from ClinicalTrials.gov.^11,12^

## Mechanism of Action and Pharmacodynamics

Xanomeline-trospium is a combination muscarinic or cholinergic agonist and antagonist; xanomeline is the muscarinic agonist agent acting within the central nervous system (CNS).^6^ Trospium is an antimuscarinic, or anticholinergic, agent that is approved for use in the United States and European Union (EU) to treat overactive bladder.^6,7^ Xanomeline exhibits high-affinity agonism at the muscarinic 1 and 4 (M_1_ and M_4_) receptors, which are predominantly located within the CNS.^6^ It binds to muscarinic 2, 3, and 5 (M_2_, M_3_, and M_5_) receptors with less affinity.^6^ Trospium exhibits antagonism of muscarinic receptors in peripheral tissues, primarily at M_3_.^6^ Trospium is peripherally acting; it is a quaternary ammonium compound that is highly polar, which makes it unable to cross the blood brain barrier and bind muscarinic receptors within the CNS.^6,7^ The primary purpose of trospium in the combination tablet is to prevent muscarinic adverse effects of xanomeline by antagonizing muscarinic receptors in the peripheral tissues.^6^ A comparison of patients taking xanomeline alone or xanomeline/trospium combination found the rate of muscarinic adverse events in the xanomeline only group was 63.6% compared with 34.3% in the combination group.^13^

Mechanistically, it is not well understood how muscarinic agonism contributes to improvement in schizophrenia symptoms. The M_1_ receptor subtype has been implicated in regulation of cognitive processes, which are known to be difficult to treat with dopaminergic antipsychotics.^14,15^ While there are several antipsychotic agents that exhibit antagonism (eg, olanzapine, quetiapine) at muscarinic receptor subtypes, clozapine has until now been unique in its partial agonism at muscarinic receptors, M_1_ and M_4_. Interestingly, clozapine is the only antipsychotic agent that reduces suicide in patients with schizophrenia, and it is recommended for patients with treatment resistant schizophrenia.^4^

All first- and second-generation agents partially agonize dopamine (DA) receptor 2 (D_2_) to some degree, with varying affinity for other DA receptor subtypes.^4^ Lack of dopaminergic receptor binding of xanomeline-trospium is notable, since this mechanism contributes to many of the adverse effects observed with both antipsychotics, including drug-induced parkinsonism and other movement disorders, hyperprolactinemia, and NMS.^4^

It is worth highlighting the complex, interconnected relationship between acetylcholine (Ach) and DA.^14^ M_4_ is an inhibitory receptor, and binding of Ach causes a downstream effect of decreased DA signaling; activation of M_4_ has been implicated in hypo-dopaminergic disorders.^14^ The N-methyl-D-aspartate (NMDA) glutamatergic pathway is also inhibited in this pathway.^14^ Decreased glutamate signaling is potentially implicated in cognitive deficits observed in schizophrenia.^14^ Interestingly, despite its implication in decreasing DA signaling, M_4_ has been identified as a target for both hypo- and hyper-dopaminergic conditions, as well as improving cognition.^14^

## Pharmacokinetics

Xanomeline is highly protein bound (95%) and has widespread distribution throughout the body tissues (volume of distribution [V_d_] = 10 800 L).^6^ Trospium also has relatively high protein binding (80%) and widespread distribution throughout the body tissues (V_d_ = 531 L).^6^ The half-life of xanomeline is 5 hours, and the half-life of trospium is 6 hours, necessitating twice daily dosing.^6^

Cytochrome P450 metabolic pathways for xanomeline include CYP2D6, CYP2B6, CYP1A2, CYP2C9, and CYP2C19.^6^ Strong inhibitors of CYP2D6 are known to increase xanomeline concentrations.^6^ The package insert includes a recommendation to monitor patients for increased frequency of adverse effects related to the medication if it is administered concomitantly with a strong CYP2D6 inhibitor medication. Pharmacogenomic variability of CYP2D6 can also impact metabolism.^6^ In intermediate metabolizers, xanomeline max concentration (C_max_) increased by 28%, and area under the curve (AUC) concentrations increased by 15%.^6^ In ultrarapid metabolizers, C_max_ and AUC both decreased by 43%.^6^ There are inadequate data to assess the impact of CYP2D6 poor metabolizer status on C_max_ and AUC for xanomeline.^6^

Xanomeline inhibits CYP3A4 and P-glycoprotein receptors in the gastrointestinal tract, but not systemically.^6^ This can cause clinically relevant drug interactions.^6^ There may also be potential for increased cholinergic or anticholinergic adverse effects when other medications that contribute to these pathways are taken concurrently. The package insert recommends monitoring for adverse effects related to concomitant use of xanomeline-trospium with CYP3A4 sensitive substrates and P-glycoprotein substrates.^6^

Trospium does not rely on the cytochrome P450 enzymatic system for metabolism—its primary metabolic pathways include ester hydrolysis and glucuronic acid conjugation.^6^ Trospium competes with other medications that utilize active tubular secretion for elimination, which can lead to increased concentration of trospium or interacting agents.^6^

## Clinical Trials

### Summary of Phase II Clinical Trial for Xanomeline-Trospium

EMERGENT-1 (NCT03697252) was a phase II, randomized, double-blind, inactive placebo-controlled 5-week trial of xanomeline-trospium in patients with a *Diagnostic and Statistical Manual of Mental Disorders*—Fifth Edition (*DSM*-5) diagnosis of schizophrenia and admitted to inpatient status for acute symptoms.^8^

The primary objective of the study was to assess change in schizophrenia symptoms using the Positive and Negative Syndrome Scale (PANSS).^8^ Appendix A describes parameters and scoring criteria for all assessments relevant to clinical trials of xanomeline-trospium, including the PANSS total and subscales. The minimum clinically important difference on the PANSS is defined as a decrease of 15 points from baseline.^16^ Appendix B describes relevant inclusion and exclusion criteria for the EMERGENT-1, EMERGENT-2, and EMERGENT-3 clinical trials.

**Figure 1.**
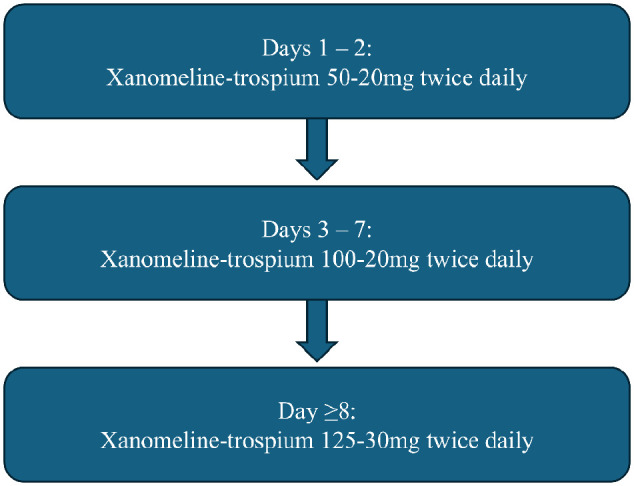
Titration schedule for xanomeline-trospium in EMERGENT-1, EMERGENT-2, and EMERGENT-3 clinical trials.

A total of 182 patients were enrolled across 12 clinical trial sites in the United States.^8^ Of 90 patients in the intervention group, 72 completed the study.^8^ The control group had 73 participants who completed the study out of 92 assigned participants.^8^ In the modified intent to treat (mITT) analysis, outcome data were reported for all participants who were randomized, received at least 1 dose of xanomeline-trospium, and had at least 1 follow-up PANSS assessment.^8^ Figure 1 describes the dosing titration used in the EMERGENT-1, EMERGENT-2, and EMERGENT-3 clinical trials; dose reduction due to adverse effects was permitted.^8-10^

Baseline characteristics were similar between the 2 cohorts.^8^ Participants were mostly black (*n* = 137, 75.3%) and male (*n* = 140, 76.9%) with a mean age of 42.5 years.^8^ Clinical global impression severity (CGI-S) is defined in Appendix A.^8^

The primary outcome was met, with least squares mean (LSM) decrease in total PANSS score from baseline of 17.4 points (± 1.749) in the intervention group compared with 5.85 points (.668) in the placebo group.^8^ Table 1 describes select secondary outcomes for the EMERGENT-1, EMERGENT-2, and EMERGENT-3 clinical trials, including movement-related safety parameters, such as the Barnes Akathisia Rating Scale (BARS), Simpson-Angus Scale (SAS), and Abnormal Involuntary Movement Scale (AIMS). There was one serious adverse event; a psychotic episode occurred in a participant in the intervention group.^8^

**Table 1.** Select Secondary Outcome Measures Comparing Intervention and Placebo Groups of EMERGENT-1, EMERGENT-2, and EMERGENT-3.

	Change in PANSS positive subscale (95% CI)^†^	Change in PANSS negative subscale (95% CI)	Change in Marder negative factor score (95% CI)	Proportion of respondents at week 5 (%)^‡^		Select safety outcomes pertaining to change in scales used to assess movement-disorders (Abnormal Involuntary Movement Scale [AIMS], Barnes Akathisia Rating Scale [BARS], Simpson-Angus Scale [SAS]; [mean ± SE])					
EMERGENT-1 ^8^	−3.2 (−4.8 to −1.7); *P* < 0.001	−2.3 (−3.5 to −1.1); *P* < 0.001	−2.5 (−3.9 to −1.2); *P* < 0.001	Intervention	Placebo	Intervention			Placebo		
				6	1 *P* = 0.15	AIMS: not assessed	BARS: −0.1 ± 1.0	SAS: −0.1 ± 0.7	AIMS: Not assessed	BARS: 0.0 ± 0.7	SAS: −0.1 ± 0.8
EMERGENT-2 ^9^	‒2.9 (–4.3 to –1.5); *P* < 0.0001	‒1.8 (–3.1 to –0.5); *P* < 0.0055	‒2.2 (–3.6 to –0.8); *P* < 0.0022	55	28 *P* < 0.0001	AIMS: −0.0 ± 0.28	BARS: −0.1 ± 1.09	SAS: −0.0 ± 0.61	AIMS: −0.0 ± 0.10	BARS: −0.1 ± 0.98	SAS: −0.1 ± 0.70
EMERGENT-3 ^10^	−3.5 (−4.7 to −2.2); *P* < 0.0001	‒0.8 (–1.9 to 0.2); *P* = 0.1224	‒0.8 (–2.1 to 0.4); *P* = 0.1957	50.6	25.3 *P* < 0.01	AIMS: −0.0 ± 0.45	BARS: −0.1 ± 0.75	SAS: −0.1 ± 0.56	AIMS: −0.0 ± 0.15	BARS: −0.1 ± 0.88	SAS: −0.1 ± 0.36

† Change in PANSS positive subscale, negative subscale, and Marder negative factor score are reported here as the absolute difference between intervention and placebo groups.

‡ In EMERGENT-1, proportion of respondents was defined as percent of participants with CGI-S score of 1 or 2 postbaseline. In EMERGENT-2 and EMERGENT-3, proportion of respondents was defined as percent of participants with ≥30% reduction in total PANSS score.

Post hoc analyses of EMERGENT-1 included evaluation of PANSS response rates,^17^ change in cognitive function,^18^ and safety and tolerability of muscarinic and antimuscarinic adverse events.^19^ Weiden et al^17^ evaluated PANSS response rates categorized by percent improvement in total score: ≥20%, ≥30%, ≥40%, and ≥50%. At week 5, 59% of the intervention group (*n* = 83) sustained a ≥20% reduction in symptoms compared with 23% of the placebo group (*n* = 87) (*P* < 0.0001, number needed to treat [NNT] = 3).^17^ Patients in all stratifications of change in total PANSS score experienced a statistically significant improvement compared with placebo, though the lowest NNT is for the ≥20% reduction cohort.^17^Change in PANSS negative symptom subscore was also assessed (defined in Appendix A).^17^ In assessment of improvement in the negative symptom domain, there was a between group difference of 2.53 points with a moderate effect size favoring the intervention (Cohen’s *d* = 0.59).^17^

Sauder et al^18^ performed an exploratory analysis assessing improvement in cognitive impairment for participants in the intervention arm of EMERGENT-1. The Cogstate Brief Battery (CBB) was administered at baseline and 5-week follow-up among patients in both the intervention and placebo arms.^18^ The CBB is a validated, computerized assessment used to measure patients’ cognitive impairment associated with schizophrenia (CIAS).^20^ The domains assessed include tests of attention, processing speed, executive function, verbal learning, and working memory, all of which were utilized as these tend to be the core deficits involved in CIAS.^18^ Participants’ baseline scores were dichotomized to either clinically significant or no/minimal cognitive impairment; clinically significant cognitive impairment was defined based on a *Z*-score decrease of one standard deviation (SD) from the normative mean, which has previously been used as a threshold difference in this population.^18^

Twenty-three participants in the intervention arm and 37 participants in the placebo arm had significant cognitive impairment at baseline.^18^ An outlier analysis assessed change in cognitive function after removing patients with excessive intraindividual variability (IIV) between scores on the CBB.^13^ It is important to note that EMERGENT-1 was not designed to assess the outcomes of this exploratory post hoc analysis.^8,18^ Excessive IIV can be due to lack of design factors controlling for variables which may impact the CBB score, such as patient comfort/mood at the time of administration, testing environment, and others.^18^

After removing outliers, authors found a statistically significant, moderate clinical improvement in CBB scores among participants with cognitive impairment at baseline in the intervention group (*n* = 20) relative to placebo (*n* = 35) (*P* = 0.03, Cohen’s *d* = 0.50), and for intraindividual scores in the intervention group (*P* = 0.01, Cohen’s *d* = 0.61).^18^ Cognitive effects were independent of improvement in PANSS scores (linear regression, *R*^2^= 0.03).^18^ There was no statistically significant difference in CBB score among the patients without cognitive impairment at baseline without removal of outliers.^18^ The practice of including an outlier analysis due to excessive IIV is well-established in examining treatment effects of mental health conditions, including schizophrenia.^18^

Correll et al^19^ analyzed adverse event data in the phase II study with a focus on cholinergic and anticholinergic adverse effects. A total of 89 patients in the intervention group and 90 patients in the placebo group received at least 1 dose of study drug and were included in post hoc analyses.^19^ Nausea (16.9%) and vomiting (9.0%) were the most common cholinergic adverse events and had median durations of 9 days and 1 day, respectively, in the intervention group.^19^

Among the frequent anticholinergic adverse events in the intervention group, constipation (16.9%) lasted 5 days on average, while dry mouth (9.0%) had a median duration of 13 days.^19^ Cholinergic and anticholinergic adverse effects were less common among patients in the placebo group: nausea (4.4%), vomiting (4.4%), constipation (3.3%), and dry mouth (1.1%).^19^ Most adverse events were rated as mild, and there were no serious adverse events related to cholinergic agonism or antagonism.^19^ Adverse events mostly occurred in the first 2 weeks of the study and were transient.^19^ Vital signs, somnolence, and metabolic parameters, such as fasting glucose, total cholesterol, and triglycerides remained stable for both cohorts.^19^ Comparatively, antipsychotics have demonstrated increases in these parameters in 6 weeks, with potential to further exacerbate metabolic syndrome when used long term.^21^

### Summary of Phase III Clinical Trials for Xanomeline-Trospium

EMERGENT-2 (NCT04659161)^9^ and EMERGENT-3 (NCT04738123)^10^ were 2 identical, phase III, multisite, randomized, double-blind, inactive placebo-controlled 5-week clinical trials of xanomeline-trospium in patients hospitalized for an acute exacerbation of schizophrenia. Both clinical trials utilized the dosing titration described in Figure 1.^9,10^ The primary endpoint in both studies was change in total PANSS score from baseline to 5-week follow-up.^9,10^ Appendix B describes relevant inclusion and exclusion criteria for the EMERGENT-2 and EMERGENT-3 clinical trials. Participants were included in the mITT analysis if they received at least 1 dose of the study drug and had a baseline and 1 or more follow-up PANSS assessment.^9,10^

EMERGENT-2 included 236 participants in the mITT analysis who were mostly black (75%), male (75%), and had a mean age of 46 years.^9^ EMERGENT-3 similarly included 256 participants in the mITT analysis, who were mostly male (74.6%) and had a mean age of 43 years; 60.9% of patients were black, and most of the remaining population was white (38.3%).^10^ All 22 EMERGENT-2 trial sites were in the United States,^9^ and the 30 trial sites in EMERGENT-3 were in the United States and Ukraine.^10^

In EMERGENT-2, participants in the intervention group had an average 21.2-point decrease in PANSS total score from baseline at 5 weeks.^9^ The LSM change in total PANSS score between the intervention and placebo cohorts at 5-week follow-up was 9.6 points.^9^ Mean change in negative symptoms based on the PANSS negative subscale was 3.4 points; using the Marder negative factor score, the mean change in negative symptoms was 4.2 points.^9^ Both scoring tools yielded a small to medium effect size (Cohen’s *d* = 0.40 [negative symptom subscale] and 0.44 [Marder negative factor score]).^9^ Other select secondary endpoints for the EMERGENT-2 clinical trial are described in Table 1.

Most patients (75%) in the intervention cohort experienced a treatment-emergent adverse event (TEAE) in EMERGENT-2, compared with 58% of the placebo cohort.^9^ Discontinuation due to adverse events was similar between the intervention (7%) and placebo (6%) groups.^9^ Four serious adverse events, unrelated to the intervention, occurred among patients taking xanomeline-trospium.^9^ Common TEAEs experienced by patients in the intervention cohort were: constipation (21%), dyspepsia (19%), nausea (19%), headache (14%), vomiting (14%), hypertension (10%), dizziness (9%), gastroesophageal reflux disease (6%), and diarrhea (6%).^9^ These adverse effects occurred less frequently in the placebo group (1%-12%).^9^

Participants in the EMERGENT-3 intervention group experienced LSM decrease in PANSS total score of 20.6 points compared with their baseline measurements.^10^ The between group difference was 8.4 points, with a moderate effect size (Cohen’s *d* = 0.60).^10^ Changes in mean score for PANSS negative symptom subscale and the Marder negative factor score were not statistically significant.^10^ Other select secondary endpoints for EMERGENT-3 are described in Table 1.

In EMERGENT-3, 70.4% of the intervention group and 50% of the placebo group experienced a TEAE.^10^ However, the rate of TEAEs leading to discontinuation was similar between the intervention (6.4%) and placebo (5.5%) arms.^10^ The most frequent TEAEs in the intervention group included nausea (19.2%), dyspepsia (16%), vomiting (16%), constipation (12.8%), hypertension (6.4%), and diarrhea (5.6%).^10^ These adverse effects occurred less frequently in the placebo group (0.8%-3.9%).^10^ There was one serious TEAE, gastroesophageal reflux disease, which occurred in the intervention group.^10^

### Pooled Analyses of EMERGENT-1, EMERGENT-2, and EMERGENT-3

Horan et al^22^ studied the pooled results of EMERGENT-1, EMERGENT-2, and EMERGENT-3 to assess the impact of xanomeline-trospium on the negative symptom subscale of PANSS given the conflicting results of EMERGENT-2 and EMERGENT-3 at 5-week follow-up. There is evidence that negative symptoms may improve independently of positive symptoms; thus, patients with predominantly negative symptomology may represent a distinct clinical group.^23^ This post hoc analysis assessed patients meeting criteria for prominent negative symptoms—moderate to severe score on PANSS Marder negative factor scale, moderate to severe scores on at least 2 core negative symptoms, and nonpredominant positive symptoms on the Mohr positive factor score (defined in Appendix A).^22^

Six hundred forty patients were included in pooled results; 64 met the criteria for prominent negative symptoms only.^21^ Of these patients, 29 received the intervention and 35 received placebo.^22^ Baseline characteristics were similar in this subpopulation.^22^ Across the overall cohort, there was a significant improvement in negative symptoms by week 5, with a moderate effect size (Cohen’s *d* = 0.42).^22^ Although a small cohort, patients with predominantly negative symptoms had a greater effect of treatment (Cohen’s *d* = 1.18) as evidenced by decrease in Marder negative factor score.^22^ In the intervention group, 76.9% of patients with prominent negative symptoms experienced ≥20% improvement in Marder negative factor score; in comparison, 55.3% of participants without predominantly negative symptoms met this threshold.^22^

Kaul et al^24^ also performed a post hoc pooled data analysis of EMERGENT-1, EMERGENT-2, AND EMERGENT-3. The purpose of this pooled analysis was to provide adequate sample sizes to increase statistical power to assess participants in stratified groups, by age, sex, race, ethnicity, nationality, baseline PANSS score, and body mass index (BMI).^24^ Six hundred forty patients were included in the mITT analysis, and a 9.9-point improvement in total PANSS score was observed in the intervention group compared with the placebo group.^24^ Patients who were in the Hispanic or Latino subgroup did not have a statistically significant improvement in PANSS total score (LSM difference = –6.6; 95% confidence interval –14.6, 1.4).^24^ The intervention had a moderate effect size, generally consistent with findings from the individual studies (Cohen’s *d* = 0.65).^24^ There was a 1.7- and 2.0-point improvement, respectively, on the negative subscale and Marder negative factor scores in the intervention group compared with the placebo group (Cohen’s *d* = 0.40).^24^

### Open-Label Extension Studies: EMERGENT-4 and EMERGENT-5

EMERGENT-4 (NCT04659174)^11^ and EMERGENT-5 (NCT04820309)^12^ are phase III open-label extension studies, with durations of 53 and 56 weeks, respectively. Patients from either group during the randomized controlled trials were eligible to enroll in the outpatient open-label studies which followed the same dose titration used in the acute setting studies.^11,12^ EMERGENT-4 has concluded and posted nonpeered-reviewed results;^11^ EMERGENT-5 (*N* = 568) concluded in May 2024 and has not released any results at the time of preparing this manuscript.^12^

Patients in EMERGENT-4 were mostly black (61.2%) or white (36.8%), male (75%), and had a mean age of 44.9 years.^11^ Among 152 patients enrolled, 35 completed the 53-week study period.^11^ The most common reasons for noncompletion were withdrawal by subject (*n* = 50), failure to adhere to protocol (*n* = 23), lost to follow-up (*n* = 20), and adverse event (*n* = 13).^11^ During the acute phase II and III 5-week studies, rates of noncompletion were much lower at approximately 3% to 7% among the intervention groups.^8-10^

More than 50% of patients experienced any TEAE in EMERGENT-4.^11^ Eight patients experienced a serious adverse event.^11^ The serious adverse events that occurred among patients who were in the intervention group during the randomized phase included injury related to fall (1.47%), anxiety (1.47%), hallucination (1.47%), and exacerbation of schizophrenia (2.94%).^11^ The serious adverse events that occurred among patients who were in the placebo group during the randomized phase included influenza infection (1.19%), exacerbation of schizophrenia (1.19%), and death (1.19%).^11^

Baseline scores for patients in the open-label extension study were the last PANSS score from the 5-week randomized studies.^11,12^ The 35 patients who completed the study were included in analysis of change in PANSS score at 52 weeks.^11^ Participants who completed the open-label phase and received xanomeline-trospium in the acute studies (*n* = 19) had an LSM improvement in total PANSS, of 9.0 points.^11^ This is clinically significant as it indicates patients who continued treatment with xanomeline-trospium in the open-label phase continued to experience symptomatic improvement with long-term use beyond the initial improvement in 5 weeks.^11^

The patients in the placebo group during the acute studies (*n* =16) had a mean improvement in total PANSS score of 23.9 points during the open-label phase.^11^ Among the patients who received the intervention during the randomized phase, 42.1% achieved a ≥30% reduction in total PANSS score from the end of the randomized phase to week 52 of the open-label phase.^11^ Among the patients who received the placebo during the randomized phase, 56.3% achieved a ≥30% reduction in total PANSS score from the end of the randomized phase to week 52 of the open-label phase.^11^

There were a large proportion of participants (77%) who did not complete the open-label extension study, EMERGENT-4.^11^ Approximately, 11% of patients did not complete the study due to a TEAE.^11^ Adherence to pharmacotherapy is known to be challenging in patients with schizophrenia, particularly in noncontrolled treatment environments.^25^ Cariprazine (Vraylar), another medication recently approved for schizophrenia, had a 50% attrition rate for its 48-week open-label phase, with 11% discontinuing treatment due to an adverse event.^26^ Additional data from the publication of EMERGENT-4 and EMERGENT-5, which have a larger sample size of 568 patients, will be helpful for further assessment of long-term safety and tolerability of xanomeline-trospium.^11,12^ Additional phase III studies, NCT05145413^27^ and NCT05304767,^28^ are currently recruiting patients to assess adjunctive use of xanomeline-trospium in a randomized controlled trial and open-label extension phase.

### Relevance to Patient Care and Clinical Practice in Comparison With Existing Drugs

Xanomeline-trospium is a novel therapeutic agent approved in the treatment of schizophrenia that exhibits its effect through muscarinic agonism; the purpose of trospium in the combination is to provide muscarinic antagonism in the periphery to prevent cholinergic adverse events.^6^

Xanomeline-trospium is available in 3 dosage strengths: 50 to 20, 100 to 20, and 125 to 30 mg, and is administered twice daily.^6^ It is recommended to be administered at least 1 hour before or 2 hours after a meal, because coadministration with food can alter C_max_ of trospium and AUC of xanomeline and trospium.^6^ Figure 1 describes the recommended dose titration. A slower dose titration can be considered in older adults and may also benefit patients who experience tolerability issues.^6^

Contraindications for xanomeline-trospium include urinary obstruction, gastric retention, hypersensitivity to any components of the medication due to risk of angioedema, and untreated narrow angle glaucoma.^6^ Additional recommendations include: avoid in patients with active biliary disease due to risk of transient biliary obstruction; use cautiously in patients with gastrointestinal obstructive disorders, ulcerative colitis, intestinal atony, or myasthenia gravis due to decreased gastrointestinal motility; monitor for anticholinergic adverse effects of the CNS, such as dizziness, confusion, hallucinations, and somnolence.^6^ Laboratory and vital sign monitoring recommendations include liver enzymes, bilirubin, and heart rate at baseline and periodically during treatment.^6^

Renal and hepatic impairment affect metabolism and elimination of xanomeline-trospium. Use of xanomeline-trospium is not recommended in patients with an estimated glomerular filtration rate (eGFR) < 60mL/min, or patients with Child-Turcotte-Pugh (CTP) class A hepatic impairment.^6^ Xanomeline-trospium is contraindicated in patients with CTP class B or C hepatic impairment.^6^ In addition, there are no data on use of xanomeline-trospium in patients who are pregnant or lactating.^6^

First- and second-generation antipsychotics utilized in the management of schizophrenia, which primarily rely on dopaminergic and serotonergic antagonism, are associated with various adverse effects that contribute to nonadherence, including metabolic syndrome and drug-induced movement disorders.^4^ Xanomeline-trospium did not appear to be associated with metabolic syndrome or acute movement-related disorders in clinical trials; long-term data will help to validate these initial findings.^8-10^ The primary adverse effects of xanomeline-trospium observed during clinical trials were cholinergic and/or anticholinergic in nature, such as nausea, vomiting, dyspepsia, constipation, dry mouth, and diarrhea.^8-10^ There were similar rates of discontinuation due to adverse effects in both the intervention and placebo cohorts of EMERGENT-1, EMERGENT-2, and EMERGENT-3.^8-10^ The safety data of xanomeline-trospium are compelling as it may indicate a good option for patients at higher risk of metabolic syndrome or who experience other intolerable adverse effects, such as drug-induced movement disorders, with antipsychotics. Treatment of Parkinsonism can contribute to pill burden and cognitive impairment due to anticholinergic burden among people with schizophrenia.^29^

Currently, no head-to-head studies providing direct comparisons of efficacy between dopaminergic antipsychotics and xanomeline-trospium are available to inform place in therapy. A systematic review and network meta-analysis^30^ was conducted to indirectly compare efficacy and safety of xanomeline-trospium with olanzapine, risperidone, and aripiprazole. A total of 33 trials and 7193 participants were included in the analysis.^30^ There was no significant difference in the magnitude of impact of the 4 active comparators on change in total PANSS score.^30^ Xanomeline-trospium was the least likely to cause weight gain and was also associated with the highest rate of all-cause discontinuation.^30^

Results from EMERGENT-4 and EMERGENT-5 will be crucial for understanding high attrition rates of xanomeline-trospium.^11,12^ From the data available thus far, prominent negative symptoms could be a compelling indication for use of xanomeline-trospium in clinical practice, although results are currently mixed.^10,17,22^ There are limited data at this time about improvements in cognitive function, but preliminary findings suggest that patients with baseline cognitive impairment may get the most benefit.^18^ One of the exclusion criteria of the EMERGENT trials was treatment resistant schizophrenia.^8-10^ Head-to-head studies of xanomeline-trospium with antipsychotics will be essential to clarify place in therapy. With currently available data, treatment experienced patients who are unable to tolerate first- and second-generation antipsychotics may be the most likely to benefit from xanomeline-trospium.

## Conclusion

Xanomeline-trospium is a novel agent that has demonstrated potential for clinically impactful improvements in schizophrenia symptoms. It appears comparable with antipsychotics in the treatment of schizophrenia among patients with moderate to severe symptoms.^12^ Xanomeline-trospium has shown promising early results on improvement in the negative symptom domain and cognitive function for patients with schizophrenia.^17,18,21^ However, it is important to keep in mind that despite the lower risk for adverse events, such as weight gain, metabolic abnormalities, movement-related disorders, hyperprolactinemia, and orthostasis, discontinuation rates were comparatively high for xanomeline-trospium versus risperidone and olanzapine;^30^ and long-term data are yet to be peer reviewed.^11,12^ Although there is currently no evidence that drug-induced movement disorders are an adverse effect of xanomeline-trospium, studies have been too short to adequately assess whether tardive dyskinesia is a possible adverse effect. Considering the modulatory effect of M_4_ on downstream dopaminergic activity, long-term studies will be useful for understanding the interplay of these effects.

While the place in therapy of xanomeline-trospium is currently uncertain, it may be most useful for individuals who are intolerant to antipsychotics or who have prominent negative symptoms. Currently, we have no data about use of xanomeline-trospium as an adjunctive agent or in patients with treatment resistant schizophrenia. Further research investigating long-term benefits and providing direct comparisons with antipsychotics is needed to clearly define the therapeutic role of xanomeline-trospium.

## Appendix A

### Definitions and scoring parameters of PANSS total score, PANSS positive subscale, PANSS negative subscale, Marder negative factor score, and CGI-S^31^

1. Scoring criteria for individual items on PANSS:
  - Absent (1)
  - Minimal (2)
  - Mild (3)
  - Moderate (4)
  - Moderate-severe (5)
  - Severe (6)
  - Extreme (7)
2. Description of PANSS total score:
  - ≤57: not ill
  - 58 to 74: mildly ill
  - 75 to 94: moderately ill
  - 95 – 115: markedly ill
  - ≥116: severely ill
3. PANSS positive subscale (possible points: 7-49)
  - Delusions (1-7)
  - Conceptual disorganization (1-7)
  - Hallucinatory behavior (1-7)
  - Excitement (1-7)
  - Grandiosity (1-7)
  - Suspiciousness/persecution (1-7)
  - Hostility (1-7)
4. PANSS negative subscale (possible points: 7-49)
  - Blunted affect (1-7)
  - Emotional withdrawal (1-7)
  - Poor rapport (1-7)
  - Passive-apathetic social withdrawal (1-7)
  - Difficulty in abstract thinking (1-7)
  - Lack of spontaneity/flow of conversation (1-7)
  - Stereotyped thinking (1-7)
5. PANSS total (possible points: 30-210)
  - PANSS positive subscale (7-49)
  - PANSS negative subscale (7-49)
  - PANSS general psychopathology subscale (16-112):
    - Somatic concern (1-7)
    - Anxiety (1-7)
    - Guilt feelings (1-7)
    - Tension (1-7)
    - Mannerisms and posturing (1-7)
    - Depression (1-7)
    - Motor retardation (1-7)
    - Uncooperativeness (1-7)
    - Unusual thought context (1-7)
    - Disorientation (1-7)
    - Poor attention (1-7)
    - Lack of judgment and insight (1-7)
    - Disturbance of volition (1-7)
    - Poor impulse control (1-7)
    - Preoccupation (1-7)
    - Active social avoidance (1-7)
6. Marder negative factor score
  - Blunted affect (negative subscale, 1-7)
  - Emotional withdrawal (negative subscale, 1-7)
  - Poor rapport (negative subscale, 1-7)
  - Passive/apathetic social withdrawal (negative subscale, 1-7)
  - Lack of spontaneity/flow of conversation (negative subscale, 1-7)
  - Motor retardation (general psychopathology subscale, 1-7)
  - Active social avoidance (general psychopathology subscale, 1-7)
7. CGI-S (1-7)
  - Normal; not at all ill (1)
  - Borderline mentally ill (2)
  - Mildly ill (3)
  - Moderately ill (4)
  - Markedly ill (5)
  - Severely ill (6)
  - Among the most extremely ill patients (7)

## Appendix B

### Inclusion and exclusion criteria for EMERGENT-1, EMERGENT-2, and EMERGENT-3

1. EMERGENT-1^8^
  - Inclusion criteria
    - Age 18 to 60 years at screening
    - Diagnosis of schizophrenia based on the DSM-5 criteria
    - Currently experiencing acute exacerbation of symptoms
      ● Onset <2 months before screening
    - PANSS total score between 80 and 120, at screening
    - Score of ≥4 (moderate) for ≥2 of the following PANSS items at screening:
      ● Delusions
      ● Conceptual disorganization
      ● Hallucinatory behavior
      ● Suspiciousness/persecution
    - If taking an injectable antipsychotic, could not have a dose of medication for at least 1.5 injection cycles before baseline visit
      ● For example, 3 or more weeks off for a 2-week cycle
    - Capable of providing informed consent
      ● Has identified reliable informant
    - CGI-S score of ≥4 at screening and baseline visits (moderately ill)
    - Body mass index must be ≥18 and ≤40 kg/m^2^
    - Females of childbearing potential and males with partners of childbearing potential must agree to use 2 forms of contraception
  - Exclusion criteria
    - >20% improvement in PANSS total score between screening and baseline
    - Any primary DSM-5 disorder other than schizophrenia within 12 months before screening
    - History or presence of clinically significant cardiovascular, pulmonary, hepatic, renal, hematologic, gastrointestinal, endocrine, immunologic, dermatologic, neurologic, or oncologic disease or any other condition that, in the opinion of the investigator, would jeopardize the safety of the subject or the validity of the study results
      ● Patients with human immunodeficiency virus (HIV), cirrhosis, biliary duct abnormalities, hepatobiliary carcinoma, and/or active hepatic viral infections may be included based on liver function test results
    - History of or high risk of urinary retention, gastric retention, narrow-angle glaucoma, irritable bowel syndrome (with or without constipation), or serious constipation requiring treatment within the last 6 months
    - Has a DSM-5 diagnosis of moderate to severe substance abuse disorder (except tobacco use disorder) within the 12 months before screening
    - Clinically significant abnormal finding on physical examination, medical history, electrocardiogram (ECG), or clinical laboratory results at screening
    - Pregnant, lactating, or less than 3 months postpartum
    - Sperm donation is not allowed for 90 days after the final dose of study drug
    - Subject has had psychiatric hospitalization(s) for >30 days (cumulative) during the 90 days before screening
    - Subject has a history of treatment resistance to schizophrenia medications defined as failure to respond to 2 adequate courses of pharmacotherapy
      ● Minimum of 4 weeks at an adequate dose, or
      ● Required clozapine within the last 12 months
    - Risk of violent or destructive behavior
    - Current involuntary hospitalization or incarceration
    - Participation in another clinical study in which the subject received an experimental or investigational drug agent within 3 months of screening
    - Subject is unsuitable for enrollment in the study according to sponsor or investigator on the basis of safety of the subject or their ability to adhere to the protocol
2. EMERGENT-2^9^
  - Inclusion criteria
    - Age 18 to 65 years at screening ^†^
    - Diagnosis of schizophrenia based on the DSM-5 criteria
    - Currently experiencing acute exacerbation of symptoms
      ● Onset <2 months before screening
      ● If already an inpatient at screening, has been hospitalized for <2 weeks^†^
    - PANSS total score at screening between 80 and 120
    - Score of ≥4 (moderate) for ≥2 of the following PANSS items at screening:
      ● Delusions
      ● Conceptual disorganization
      ● Hallucinatory behavior
      ● Suspiciousness/persecution
    - Capable of providing informed consent
      ● Has identified reliable informant
    - CGI-S score of ≥4 at screening and baseline visits (moderately ill)
    - Body mass index must be ≥18 and ≤40 kg/m^2^
    - Females of childbearing potential and males with partners of childbearing potential must agree to use 2 forms of contraception
    - Must discontinue lithium therapy for ≥2 weeks before baseline visit ^†^
    - Must discontinue oral antipsychotic medications for ≥5 half-lives or 1 week (whichever is longer) before baseline visit ^†^
    - If taking an injectable antipsychotic, could not have a dose of medication for ≥12 weeks ^†^
      ● ≥24 weeks for Invega Trinza^†^
    - Subject is willing and able to be confined to an inpatient setting for the study duration, follow instructions, and comply with the protocol requirements ^†^
  - Exclusion criteria
    - >20% improvement in PANSS total score between screening and baseline
    - Any primary DSM-5 disorder other than schizophrenia within 12 months before screening
    - Subjects who are newly diagnosed or are experiencing their first treated episode of schizophrenia ^†^
    - History or presence of clinically significant cardiovascular, pulmonary, hepatic, renal, hematologic, gastrointestinal, endocrine, immunologic, dermatologic, neurologic, or oncologic disease or any other condition that, in the opinion of the investigator, would jeopardize the safety of the subject or the validity of the study results
      ● Patients with human immunodeficiency virus (HIV), cirrhosis, biliary duct abnormalities, hepatobiliary carcinoma, and/or active hepatic viral infections may be included based on liver function test results
    - History of or high risk of urinary retention, gastric retention, narrow-angle glaucoma, irritable bowel syndrome (with or without constipation), or serious constipation requiring treatment within the last 6 months
    - Has a DSM-5 diagnosis of moderate to severe substance abuse disorder (except tobacco use disorder) within the 12 months before screening
    - Risk for suicidal behavior during the study as determined by the investigator’s clinical assessment and Columbia-Suicide Severity Rating Scale (C-SSRS) ^†^
    - Clinically significant abnormal finding on physical examination, medical history, ECG, or clinical laboratory results at screening
    - Pregnant, lactating, or <3 months postpartum
    - Sperm donation is not allowed for 90 days after the final dose of study drug
    - Subject has had psychiatric hospitalization(s) for >30 days (cumulative) during the 90 days before screening
    - Subject has a history of treatment resistance to schizophrenia medications defined as failure to respond to 2 adequate courses of pharmacotherapy
      ● Minimum of 4 weeks at an adequate dose
      ● Or required clozapine within the last 12 months
    - Risk of violent or destructive behavior
    - Current involuntary hospitalization or incarceration
    - Participation in another clinical study in which the subject received an experimental or investigational drug agent within 3 months of screening
    - Subject is unsuitable for enrollment in the study according to Sponsor or investigator on the basis of safety of the subject or their ability to adhere to the protocol
    - Positive test for coronavirus (COVID-19) within 2 weeks before screening and at screening^†^
    - Subjects with extreme concerns relating to global pandemics, such as COVID-19, that preclude study participation^†^
    - Subjects with prior exposure to KarXT^†^
    - Subjects who experienced any adverse effects due to xanomeline or trospium
3. EMERGENT-3^10^
  - Inclusion criteria
    - Age 18 to 65 years at screening ^†^
    - Diagnosis of schizophrenia based on the DSM-5 criteria
    - Currently experiencing acute exacerbation of symptoms
      ● Onset <2 months before screening
      ● If already an inpatient at screening, has been hospitalized for <2 weeks ^†^
    - PANSS total score at screening between 80 and 120
    - Score of ≥4 (moderate) for ≥2 of the following PANSS items at screening:
      ● Delusions
      ● Conceptual disorganization
      ● Hallucinatory behavior
      ● Suspiciousness/persecution
    - Capable of providing informed consent
      ● Has identified reliable informant
    - CGI-S score of ≥4 at screening and baseline visits (moderately ill)
    - Body mass index must be ≥18 and ≤40 kg/m^2^
    - Females of childbearing potential and males with partners of childbearing potential must agree to use 2 forms of contraception
    - Must discontinue lithium therapy for ≥2 weeks before baseline visit^†^
    - Must discontinue oral antipsychotic medications for ≥5 half-lives or 1 week (whichever is longer) before baseline visit ^†^
    - If taking an injectable antipsychotic, could not have a dose of medication for ≥12 weeks ^†^
      ● ≥24 weeks for Invega Trinza ^†^
    - Subject is willing and able to be confined to an inpatient setting for the study duration, follow instructions, and comply with the protocol requirements ^†^
  - Exclusion criteria
    - >20% improvement in PANSS total score between screening and baseline
    - Any primary DSM-5 disorder other than schizophrenia within 12 months before screening
    - Subjects who are newly diagnosed or are experiencing their first treated episode of schizophrenia ^†^
    - History or presence of clinically significant cardiovascular, pulmonary, hepatic, renal, hematologic, gastrointestinal, endocrine, immunologic, dermatologic, neurologic, or oncologic disease or any other condition that, in the opinion of the investigator, would jeopardize the safety of the subject or the validity of the study results
      ● Patients with human immunodeficiency virus (HIV), cirrhosis, biliary duct abnormalities, hepatobiliary carcinoma, and/or active hepatic viral infections may be included based on liver function test results
    - History of or high risk of urinary retention, gastric retention, narrow-angle glaucoma, irritable bowel syndrome (with or without constipation), or serious constipation requiring treatment within the last 6 months
    - Has a DSM-5 diagnosis of moderate to severe substance abuse disorder (except tobacco use disorder) within the 12 months before screening
    - Risk for suicidal behavior during the study as determined by the investigator’s clinical assessment and Columbia-Suicide Severity Rating Scale (C-SSRS)^†^
    - Clinically significant abnormal finding on physical examination, medical history, ECG, or clinical laboratory results at screening
    - Pregnant, lactating, or <3 months postpartum
    - Sperm donation is not allowed for 90 days after the final dose of study drug
    - Subject has had psychiatric hospitalization(s) for >30 days (cumulative) during the 90 days before screening
    - Subject has a history of treatment resistance to schizophrenia medications defined as failure to respond to 2 adequate courses of pharmacotherapy
      ● Minimum of 4 weeks at an adequate dose
      ● Or required clozapine within the last 12 months
    - Risk of violent or destructive behavior
    - Current involuntary hospitalization or incarceration
    - Participation in another clinical study in which the subject received an experimental or investigational drug agent within 3 months of screening
    - Subject is unsuitable for enrollment in the study according to Sponsor or investigator on the basis of safety of the subject or their ability to adhere to the protocol
    - Positive test for coronavirus (COVID-19) within 2 weeks before screening and at screening ^†^
    - Subjects with extreme concerns relating to global pandemics, such as COVID-19, that preclude study participation ^†^
    - Subjects with prior exposure to KarXT ^†^
    - Subjects who experienced any adverse effects due to xanomeline or trospium

^†^Change from EMERGENT-1 criteria.
